# Artificial intelligence and suicide prevention: A systematic review

**DOI:** 10.1192/j.eurpsy.2022.8

**Published:** 2022-02-15

**Authors:** Alban Lejeune, Aziliz Le Glaz, Pierre-Antoine Perron, Johan Sebti, Enrique Baca-Garcia, Michel Walter, Christophe Lemey, Sofian Berrouiguet

**Affiliations:** 1 URCI Mental Health Department, Brest Medical University Hospital, Brest, France; 2 Mental Health Department, French Polynesia Hospital, FFC3+H9G, Pirae, French Polynesia; 3 Departamento de Psiquiatria, IIS-Fundación Jiménez Díaz, Madrid, Spain; 4 EA 7479 SPURBO, Université de Bretagne Occidentale, Brest, France; 5 SPURBO, IMT Atlantique, Lab-STICC, UMR CNRS 6285, F-29238, Brest, France; 6 LaTIM, INSERM, UMR 1101, Brest, France

**Keywords:** Artificial intelligence, machine learning, neural network, suicide prevention

## Abstract

**Background:**

Suicide is one of the main preventable causes of death. Artificial intelligence (AI) could improve methods for assessing suicide risk. The objective of this review is to assess the potential of AI in identifying patients who are at risk of attempting suicide.

**Methods:**

A systematic review of the literature was conducted on PubMed, EMBASE, and SCOPUS databases, using relevant keywords.

**Results:**

Thanks to this research, 296 studies were identified. Seventeen studies, published between 2014 and 2020 and matching inclusion criteria, were selected as relevant. Included studies aimed at predicting individual suicide risk or identifying at-risk individuals in a specific population. The AI performance was overall good, although variable across different algorithms and application settings.

**Conclusions:**

AI appears to have a high potential for identifying patients at risk of suicide. The precise use of these algorithms in clinical situations, as well as the ethical issues it raises, remain to be clarified.

## Introduction

Overall mortality from suicide is currently about 700,000 deaths per year [1]. Suicide and suicidal behavior are a public health concern. Among people surviving a suicide attempt, about one-third come to the emergency department for help [2]. Suicide risk assessment of these patients is a daily challenge in psychiatric practice. The personal and family history, particularly of suicide attempts, plays a major role in assessing suicide risk. Personal history of suicide attempt is the most significant factor in the risk of death by suicide [2]. Patients with severe mental illness, in particular mood disorder, borderline personality disorder, and anorexia nervosa, are more likely to attempt suicide [3,4]. They are at higher risk of recurrence in the years following the first attempt [5]. Among patients who survived a suicide attempt, a study found that certain subgroups (alcohol consumption, personality disorder, and young age) were also more likely to attempt suicide again, and others were more likely to die by suicide after a first attempt (elderly patients) [6]. In clinical practice, particular attention must also be given to the period of discharge from the care services. The risk appears to be increased within the 2 weeks following discharge from the hospital [2].

Since the incidence of suicide and suicide attempts remains high [1,7,8], we need new approaches to identify and manage patients at high risk of suicide. The current suicide risk assessment methods are based on questioning and therefore subject to subjectivity. Their accuracy and predictive value are limited [9]. Several scales can be used in the suicide risk assessment, but their accuracy seems insufficient [10]. In their meta-analysis, Franklin et al. [11] found that the ability to predict suicide had not improved over the past 50 years. The ability to predict a suicide attempt lack accuracy. Advances in suicide risk assessment are needed [11].

Artificial intelligence (AI) and machine learning (ML) have emerged as ways to improve risk detection [12]. These techniques require a large database (*big data*) to extract a patient’s profile or significant risk factors [13]. AI platforms can identify patterns in the dataset to generate risk algorithms and determine the effect of risk and protective factors on suicide [9]. AI has already been successfully applied to other medical disciplines (imagery, pathology, dermatology, etc.). In these disciplines, it is already faster than medical experts, with equivalent accuracy, for the diagnosis of certain pathologies. Although the diagnostic accuracy never reaches 100%, this technology combined with the skills of the clinician could greatly improve overall performance [14]. In psychiatry, AI could be used for diagnostic purposes, to support daily patient assessment or drug prescription. Beyond its medical value, AI could show a clear economical benefit [12].

In their systematic literature review, Burke et al. identified three main goals of ML studies in suicide. The first was to improve the accuracy of risk prediction, the second was to identify important predictors and the interactions between them, and the third was to model subgroups of patients [15]. The studies that focused on improving suicide risk prediction capabilities suggested a high predictive potential for this technology [8,12,16,17]. At an individual level, AI could allow for better identification of individuals in crisis and appropriate intervention. At the population level, the algorithm could find groups at risk [9] and individuals at risk of suicide attempt in these groups [18]. Decision support tools could also allow for a more accurate assessment of suicidal risk in situations where the patient denies suicidal ideation [19].

In clinical practice, this technology could help the clinician to more effectively identify patients at risk of suicide, with the goal of improving predictive abilities for suicide. Further studies are required to validate this tool and apply it to clinical practice [8,20].

In their narrative review published in 2020, D’Hotman et al. [21] conclude that AI has a high potential in suicide risk prediction, albeit with ethical reservations regarding the use of individual data. To clarify the potential of this technology, we conducted a systematic review of the literature including clinical studies using AI to assess suicide risk. To our knowledge, this is the first systematic review on this subject. The objective of this review is to evaluate the potential of AI in predicting individual suicide risk and identifying individuals at risk of suicide attempt in a population.

## Material and Methods

We used PRISMA criteria (Preferred Reporting Items for Systematic reviews and Meta Analyses) to identify, select, and critically assess relevant studies while minimizing bias.

### Search strategy

We went through the bibliographic databases PubMed, SCOPUS, and EMBASE until April 2020. We based the keywords list on two fields: suicide and AI. A search strategy was built by using the Booleans operator “AND” and “OR” and applied to titles and abstracts. The keywords and the search strategy were (suicid*[Title]) AND (artificial intelligence [Title/Abstract] OR AI[Title/Abstract] OR neural network[Title/Abstract] OR deep learning[Title/Abstract] OR machine learning[Title/Abstract]) in any language, but referenced in the selected databases. To limit the selection bias, we did not apply any restriction in terms of the type of article or population. Studies that were not written in English were excluded.

### Study selection

We included clinical trials and observational studies. The primary objective was to collect studies using AI to predict individual suicide risk or for identification of individuals at risk of suicide in a population. Studies were selected by two independent authors, Alban Lejeune and Sofian Berrouiguet. We excluded studies reviewing literature or studying the theoretical applications of AI without any final results. We also excluded studies studying computer programs or smartphone applications that did not use AI to assess suicide risk.

### Data collection process

Data were extracted from each article independently by using a standard form. The following information was collected: the main author’s name and country of origin, year of publication, population, technology used, inclusion/ exclusion criteria, main objective, method, main endpoint, results, and authors’ conclusion.

## Results

### Flow chart

**Figure 1.**
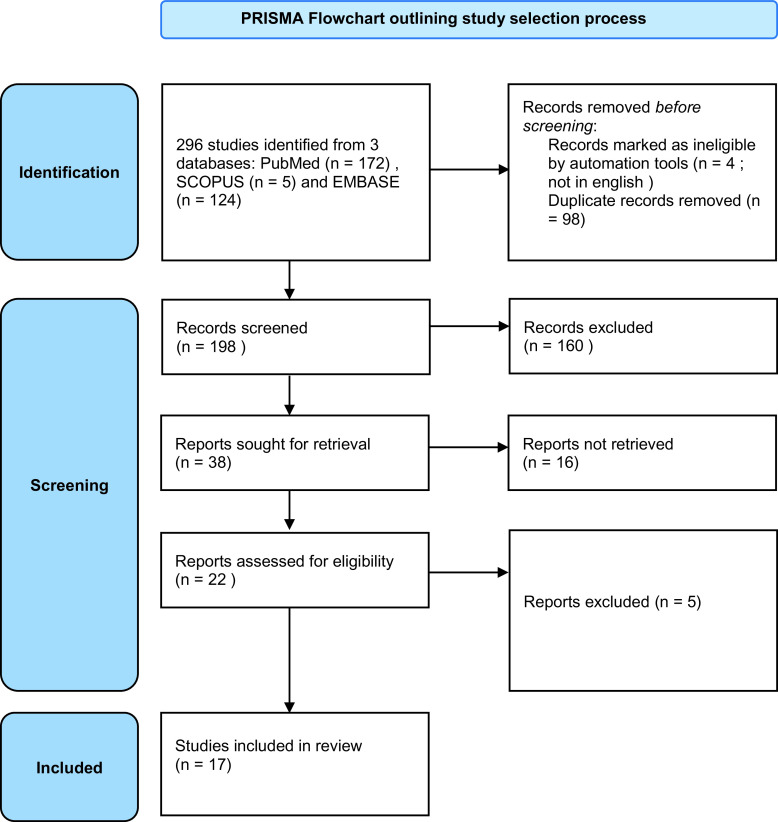
PRISMA flowchart outlining the study selection process.

Figure 1 shows the PRISMA flow chart, summarizing the steps of the review. The initial search identified 296 studies. Based on the titles and abstracts, we excluded 249 studies. We downloaded the 47 remaining studies for full-text review, following which we excluded an additional 30 studies. We analyzed the 17 remaining studies that matched the inclusion criteria.

### Authors, year of publication, and country of origin

**Figure 2.**
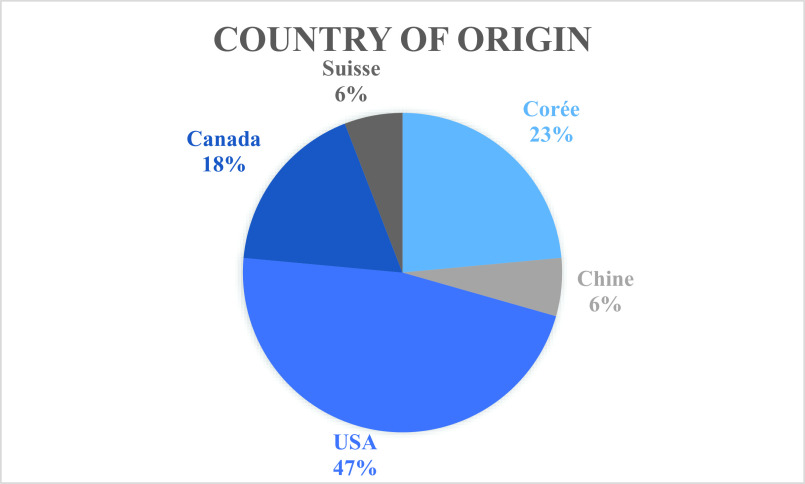
Included studies by country of origin.

**Figure 3.**
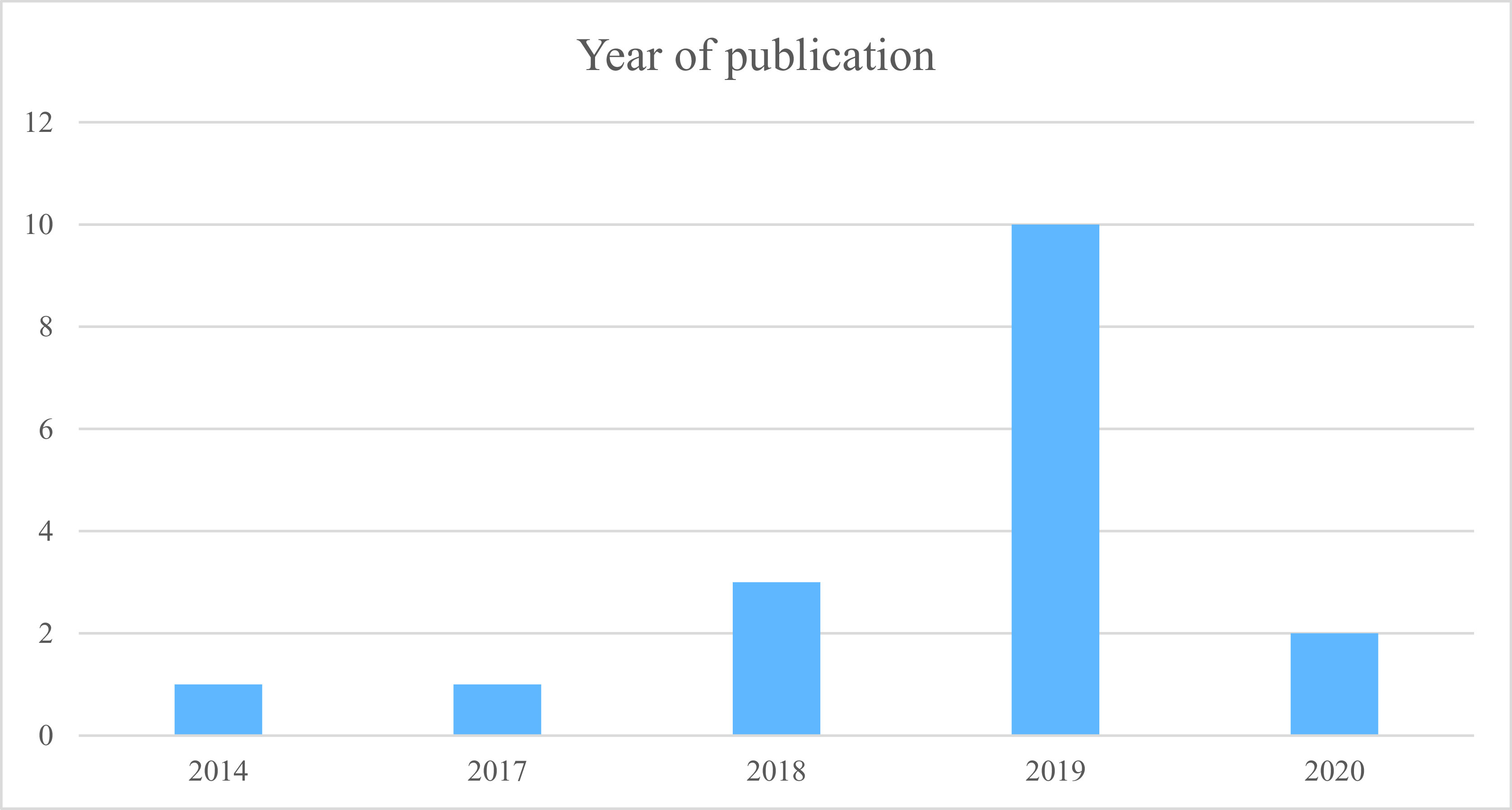
Number of studies included by year of publication.

Included studies were mainly conducted in the USA (8/17, 47%), Korea (4/17, 24%), and Canada (3/17, 18%; Figure 2). Among the 17 included studies, three were written by the team of Sanderson et al. Included studies were published between 2014 and April 2020. The majority of studies were published in 2019 (10/17, 59%), three studies were published in 2018 (3/17, 18%), two studies in 2020 (2/17, 12%), one study in 2017, and one study in 2014 (Figure 3).

### Studies design, populations, and sample size

**Figure 4.**
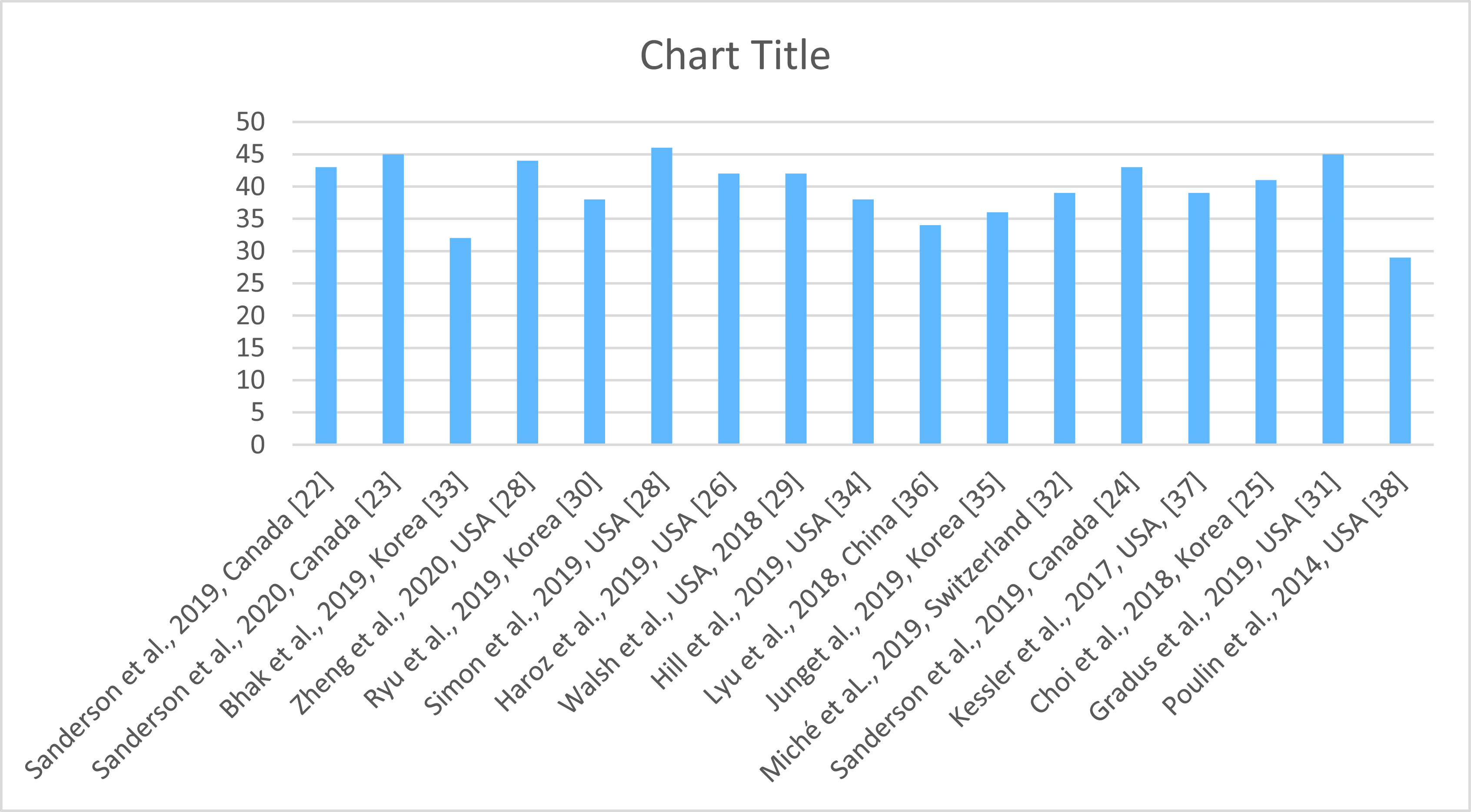
PRISMA quality assessment of the included studies.

Regarding the design of included studies, 13 studies had a retrospective design and 4 studies had a prospective design. Samples’ size varied between 182 and 19,061,056. Four studies used a sample size inferior to 10,000. Six studies used a sample size between 1,000 and 10,000 and seven studies used a sample size greater than 10,000. The studies were conducted on the general population in seven studies (47%), on adult patients in two studies (13%), on teenagers or young adults in four studies (27%), on an ethnic group or a particular subgroup in two studies (13%) and on militaries in two studies (13%). In the PRISMA quality assessment, the included studies obtained heterogeneous scores. Their scores ranged from 29 to 46 (see Supplementary File S2 and Figure 4).

### Technologies used

**Figure 5.**
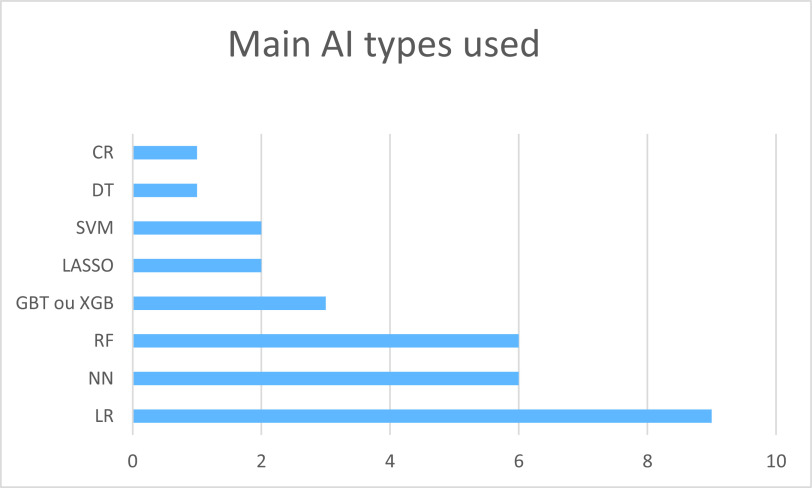
Main AI types used. *Abbreviations*: AI, artificial intelligence; CR, cox regression; DT, decision tree; LR, logistic regression; NN, neural network; RF, random forest; SVM, support vector machine; XGB/GBT, extreme gradient boosting/gradient boosted tree.

Included studies used one or several AI technologies. The main algorithms used were the logistic regression (LR; 9/17, 53%), the random forest (6/17, 35%), the gradient-boosting algorithms (3/17, 18%), the LASSO (2/17, 12%), and the support vector machine (SVM; 2/17, 12%). Six studies used at least one type of neural network (NN; 35%). Most studies used cross validation (15/17, 88%; Figure 5). The most used ML feature was supervised learning. None of the included studies used the data augmentation technique.

### Objective of the studies and performance of algorithms

#### Main results

The two fields of interest were the suicide risk prediction and the identification of people at risk in a given population, using one or more AI technologies. Among the 17 studies included, 13 studies (76%) mainly aimed at predicting the individual suicide risk. Four studies (24%) mainly aimed at identifying individuals at risk in a population.

**Figure 6.**
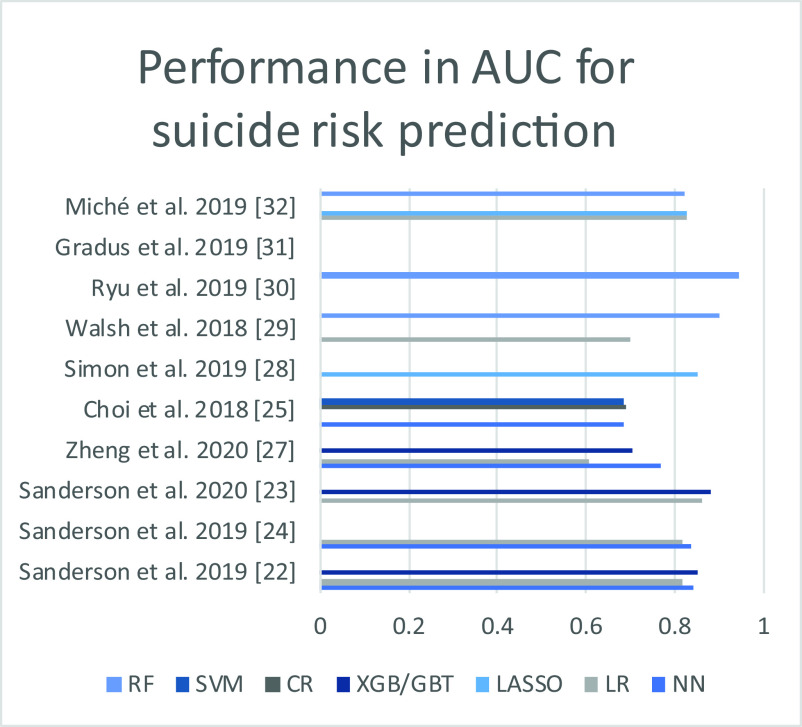
Performance in AUC of the different algorithms, based on the studies included in Table 1. *Abbreviatioins*: AUC, area under the curve; BN, Bayesian network; DT, decision tree; LR, logistic regression; NN, neural network; RF, random forest; XGB/GBT, extreme gradient boosting/gradient boosted tree.

In the prediction of individual suicidal risk, the different studies found an area under the curve (AUC) performance between 0.604 and 0.947. NNs and boosted gradient algorithms appeared to perform best in the studies that used them (see Table 1 and Figure 6). The performance of the different algorithms was mainly informed with the parameter AUC (Supplementary File S1). Four studies informed other parameters (sensibility; sensitivity; accuracy; and true and false predictive value). Among included studies, three studies were conducted in Canada by Sanderson et al. [22–24]. These studies focused on comparing the relative performance of different algorithms as well as NNs in predicting suicidal risk. It appears that NNs and gradient boosted algorithms (XGBs) seem to bring a significant improvement compared to LR models. In their last article [23], an XGB algorithm is compared to LR models regarding the prediction of suicide risk during the 90 days following the discharge from an emergency department. The XGB model then provides superior discrimination and calibration, with an accuracy that could allow clinical application (AUC 0.88). A Korean team [25] has however obtained a lower performance with a NN than with a cox regression (CR) or SVM algorithm.

**Table 1.** Performance in the prediction of suicide risk with the main algorithms, expressed in AUC, in studies in which this value was informed.

	NN	LR	LASSO	XGB/GBT	CR	SVM	RF	Cross validation
Sanderson et al. (2019) [22]	0.842	0.818		0.849				Yes
Sanderson et al. (2019) [24]	0.8352	0.8179						Yes
Sanderson et al. (2020) [23]		0.86		0.88				Yes
Zheng et al. (2020) [28]	0.769	0.604		0.702				Yes
Choi et al. (2018) [25]	0.683				0.688	0.687		Yes
Simon et al. (2019) [29]			0.85					Yes
Walsh et al. (2018) [27]		0.7					0.9	No
Ryu et al. (2019) [30]							0.947	Yes
Gradus et al. (2019) [31]							080–0.88	Yes
Miché et al. (2019) [32]		0.828	0.826				0.824	Yes

*Abbreviations*: AUC, area under the curve; BN, Bayesian network; CR, cox regression; LR, logistic regression; NN, neural network; RF, random forest; SVM, support vector machine; XGB/GBT, extreme gradient boosting/gradient boosted tree.

In the identification of at-risk individuals in a specific population, the results were presented in sensitivity/specificity/precision. The AUC was reported in one article [26].

Most studies (15/17) used cross validation to prevent overfitting. One team [27] used bootstrapping with optimism adjustment instead of cross validation. The predictive models were trained and tested on all study data. The optimism of the model was estimated by repeating the same steps on bootstrapped replicates, and by subtracting from the performance the summed difference between bootstrapped replicates.

#### Data used

Data used to supply ML models were mainly those from health systems. A study [29] compared a model using only data from patient records from a health system database with several models using the same data combined with additional data, particularly clinical data (sociodemographic data, results from a questionnaire, data from an index consultation). The aim was to predict suicidal risk in the 90 days following a consultation for suicidal ideation. This study found approximately equivalent performance between the models (AUC 0.843 vs. 0.850) [29]. Data collected during the medical visit provided a statistically significant improvement in the prediction of suicidal risk, but with a low effect size. A Korean team [30] sought to identify patients at risk of suicide among those who expressed suicidal ideations in a self-administered questionnaire, by analyzing retrospective data from a national database. This team reported good overall performance, with an accuracy of 88.9% and an AUC of 0.947.

Among the included studies, four studies used a prospective design. Zheng et al. [28] used a deep NN to prospectively predict the one-year suicide risk and identify people at risk of suicide. The data used were solely from a health database. Performance was acceptable with an AUC of 0.769 (95% CI: 0.721–0.817). This deep learning model significantly improved performance, compared to other algorithms tested on the same cohort in this study (XGB: AUC 0.702; LR: AUC 0.604). A study [33] sought to prospectively identify patients with a major depressive disorder and suicidal patients by analyzing blood markers (methylomes and transcriptomes) on a small sample. Their random forest model found an accuracy of 92.6% to distinguish suicidal from characterized depressive episodes and an accuracy of 86.7% to distinguish suicidal from control subjects. Miché et al. also prospectively assessed the risk of suicide attempts in adolescents and young adults [32]. Hill et al. [34] sought to prospectively identify adolescents who would attempt suicide from a large sample.

#### Outcomes in teenagers and young adults

Four studies have investigated the prediction of suicidal risk or the identification of at-risk patients in adolescents and young adults. Miché et al. [32] studied four ML algorithms in suicide attempt risk assessment. They found close performance between these (AUC between 0.824 and 0.829) for patients aged from 14 to 24 years. The highest AUC was obtained with ridge regression. Hill et al. [34] sought to identify patients at risk for suicide attempts among a large cohort of 4,834 teenagers during 12 months. Two classification trees had reached a higher risk prediction, with a sensitivity/ specificity profile of 69.8%/85.7% for the first and 90.6%/70.9% for the second. A Korean study [35] aimed to retrospectively identify at-risk patients in a national database, including 59,084 teenagers. All the models used had an accuracy between 77.5 and 79%, comparable to the accuracy of the LR (77.9%). The most accurate model was XGB (79%) and the least accurate model in this study was an artificial neural network (ANN) (77.5%). In 2018, Walsh et al. [27] studied the prediction of adolescent suicide attempts through a retrospective longitudinal cohort. Several time periods were analyzed (from 1 week to 2 years). A random forest model was compared to LR. Performance was good, without the need for a face-to-face meeting (AUC approximately between 0.8 and 0.9 depending on the time window chosen, with the AUC being better the more imminent the suicide attempt was).

#### Use of AI in specific populations

Five publications studied specific populations. Haroz et al. [26] tried to identify at-risk patients among a Native American community during the 24 months following an initial suicide attempt. With four ML algorithms, they obtained an AUC between 0.81 (decision tree) and 0.87 (ridge regression). In comparison, the AUC for previous suicide attempt was 0.57. Lyu et al. [36] used a backpropagation NN to predict suicide risk in rural China inhabitants, with a total coincidence rate of 84.6%. Kessler et al. [37] aimed to identify American veterans at high risk of suicide. They found a similar sensibility between algorithms for detecting at-risk veterans. The best-performing model in this study was the Bayesian additive regression tree algorithm, with 28% of suicides included in the 5% of veterans detected as a highest risk by the algorithm. Poulin et al. [38] sought to identify veterans at risk for suicide through analysis of each patient’s medical observations. With a supervised ML algorithm retrospectively analyzing medical records of veterans who died by suicide, they obtained an accuracy of 67–69%.

Gradus et al. [31] sought to predict suicide risk according to gender by using several Danish databases, thus including a very large sample of patients. With a random forest algorithm, they obtained a good predictive performance of suicide risk (AUC of 0.80 in men and 0.88 in women).

## Discussion

### Main results

Our review shows an exponential gain in interest in the application of AI in the field of suicide prevention. The selected studies were all published between 2014 and 2020. Several studies have been published since the end of this review [39–41] which demonstrates the interest in this subject. A Korean team conducted a meta-analysis in April 2021 to directly compare the predictive capabilities of four leading suicide theories to ML [42]. This growing interest is in line with the major development of AI, which is currently one of the main emerging technologies.

This article has provided an inventory of studies using AI to assess individual suicide risk and to identify patients at high risk of suicide. These studies suggest that AI could be an effective technology for this purpose, with several algorithms used and reproducible results in different populations. This review is, to our knowledge, the first systematic review on this topic.

### Limitations of this study

This preliminary study has several limitations. Firstly, it includes a low number of studies. The number of studies using AI for suicide prevention is increasing exponentially, but the number of published studies is still low to date. However, this study does not aim at a precise evaluation of the performance of AI in a given situation, but at an assessment of the potential of this technology. We have listed the performance of the different algorithms tested to better assess this potential. Secondly, the selected studies were published in a small number of countries. Most studies (88%) were published in the USA, Canada, or Korea. These countries have significant health databases, and AI could be more challenging to implement in countries or regions where patient data is less accessible or less accurate [43].

The studies included used mostly retrospective data. The performance of these algorithms may be lower in clinical practice, in heterogeneous populations, and with prospective data. The performance of AI in clinical situations is still unknown at this time and remains to be clarified.

Finally, some studies did not use a cross-validation technique to limit overfitting. Their results may therefore have been over-optimistic. However, only two of the included studies did not use cross validation.

### Feasibility, recommendation for use of AI

The included studies that used AI to predict suicidal AAP found overall good performance on the most commonly used algorithms (LR, XGB/GBT, NN, and RF) with an AUC approximately between 0.8 and 0.9 (see Table 1 and Figure 5). The data required to achieve such a performance is probably less voluminous than initially assumed. Some of the included studies indeed found a good performance with the use of health system data alone [25,29]. It is possible that the application of AI to the health system data already collected would be sufficient to allow a significant advance in the prediction of suicide risk. It could be a first step towards the use of this technology. The use of complex algorithms is likely to lead to better performance, but some simpler algorithms, such as LR, have relatively close performance at present. These algorithms are probably easier and cheaper to implement in the health system.

If the performance of this technology is similar in clinical practice, it could lead to a more accurate prediction of suicidal risk and thus to significant changes in the management of patients at risk of suicide.

### Patients’ data collection: ethical reflection

AI raises ethical concerns within the medical community [44,45], especially regarding the use of data, the place of the practitioner in care, and ethical and medico-legal responsibility.

Patients’ data already have an important place in the assessment and management of suicidal behavior. Practitioners use patients’ data, such as their personal and familial history, their care history, their socio-demographic, and ethnical characteristics, to evaluate the risk of suicide attempt. AI can optimize the analysis of these data and thus yields a better efficiency. The application of AI to health data will require robust cyber security, as well as a clear legal framework.

AI is complementary to the medical assessment and does not replace it. Optimal performance will probably be reached through the proper use of AI by the physician, with holistic patient care. The doctor–patient relationship will remain essential in patient care. AI could raise a responsibility problem. This question remains unresolved. AI could allow an information gain. The clinicians will have more elements to assess a situation and lead his management. It seems to us that the responsibility will always go to the clinician, once he is informed of the performance and limitations of this technology in precise clinical situations. The physician will then be able to organize personalized care. The medical profession is already proceeding in this way for other technologies used in medicine. Current data suggest an interesting performance of AI in suicide prevention and justify a more precise exploration of this tool.

## Conclusion

AI is increasingly being used in suicide research, with a recent increase in the number of studies published. This technology may allow a significant evolution in suicide risk assessment, with a more accurate and reliable assessment than with present methods. This tool is likely to become more accessible in the coming years. AI is already being used successfully in other medical disciplines. This technology will probably have its place as a complement to existing tools in suicide prevention. However, AI is not yet usable in clinical practice. The performance presented in this article is based on retrospective data. The performance of AI in clinical practice remains unknown. Further studies are required to clarify the value of this technology in suicide risk assessment, including prospective studies in clinical application.

## Data Availability

All the studies included in this paper are available on the quoted databases (PubMed, EmBASE, and SCOPUS). Extracted data from the studies are available in Supplementary File S2.

## Supplementary Materials

To view supplementary material for this article, please visit http://doi.org/10.1192/j.eurpsy.2022.8. click here to view supplementary material
