# Supplementary material for: Artificial intelligence and suicide prevention: A systematic review
Source: Eur Psychiatry. 2022 Feb 15;65(1):e19. doi: 10.1192/j.eurpsy.2022.8 (PMC8988272; doi:10.1192/j.eurpsy.2022.8)
Supplement: Supplementary file 1 [file S0924933822000086sup001.zip › S0924933822000086sup001.docx]

**Supplementary file 1****. Focus on artificial intelligence algorithms mentioned in this article**

*Supervised algorithms:*

Supervised learning algorithms allow patterns correlated to a result to be determined in a dataset^1^. These patterns can be used to classify risk ^2^. The supervised algorithms are separated into two categories: regression and classification.

*Classification algorithms* allow data to be classified into separate categories. *Decision tree* (DT), *support vector machine* (SVM) and *random forest* (RF) can perform classification tasks^2^.

*Regression algorithms* are used to predict quantitative data. *Logistic regression* (LR) and *LASSO regresssion* are part of this class.

*Unsupervised algorithms:*

With unsupervised learning algorithms, patterns are not correlated to a result. These algorithms will attempt to determine patterns or clusters within a dataset in order to categorize them into groups^2,3^. The k*-means algorithm* is part of this class.

*Neural Networks:*

Artificial neural networks are algorithms that have been built in reference to the cortical neural structure. They are powerful artificial intelligence (AI) tools. They can perform supervised or unsupervised tasks. Neural networks (NN) are organized in a succession of layers. Each layer has its input on the output of the previous one. There are one input layer, one or several hidden layers, and one output layer. The input neurons receive the raw information. These neurons (or nodes) are connected to a varying number of hidden layers. Information travels from the input neurons to the hidden layers before arriving at the outcome layer where the final decision is made^3^.

In a neural network, each layer functions differently. The input and output units operate linearly and the hidden layers operate non-linearly. A *feedforward neural network* (FNN), the simplest form of NN, has a single hidden layer. It allows the network to have a flexible approximation of a function linking data to a desired result. A FNN can have an approximation of any continuous function with any degree of precision by playing on the number of nodes of the different layers^4^. Neural networks can be categorized as a *deep neural network* (DNN) if they have more than one hidden layer. A "deeper" architecture of the neural network can increase precision with a fixed number of parameters. With DNN, the deep parameters of the network are determined on the basis of experience (i.e. secondarily to the analysis of the data previously provided). DNN can learn from experience. This type of AI requires significant computing power and large databases^4^. Deep neural networks have complex settings and are more prone to overfitting^3^.

*Overfitting and crossvalidation:*

Overfitting means fitting an AI model on data noise or error instead of the actual relationship. Overfitting is either due to having a small data sample or too many parameters compared to the data^1^. Crossvalidation is a technique for reducing overfitting. With this technique, the dataset is split into several groups, themselves divided into training data and validation data. For each group, the statistical model is therefore trained and then validated by a different dataset. This technique reduces the risk of having an overoptimistic estimate^5^.

**Examples of the use of machine learning in medicine and psychiatry:**

AI has multiple potential applications in medicine and psychiatry^6^. The supervised algorithms can be used, for example, to recognize the patterns on an EKG and to link them to a diagnosis. In radiology, a supervised algorithm could recognize a lung tumor on a CT scan. These algorithms could allow assisted diagnosis^2^. In our article, supervised algorithms are used to assess suicide risk. Unsupervised algorithms could allow a more precise approach of complex and clinically heterogeneous diseases^2^. For example, an American team^7^ used a clustering algorithm to classify chronic psychotic disorders. Deep neural networks can achieve precise results when assigned to complex tasks. They could recognize pictures and help to the diagnosis of melanoma^3^.

A research team used a DNN to diagnose early-stage Alzheimer's dementia on MRI data with excellent accuracy. Neural networks have also been studied to predict response to psychotropic treatment^8^. A Chinese study used an NN to discriminate schizophrenic patients from healthy controls on MRI data^9^.

**Performance evaluation criteria:**

Several parameters can be used to evaluate the performance of machine learning algorithms. Sensitivity is the proportion of true positives correctly identified by the test. Specificity is the proportion of correctly identified true negatives. Precision is the proportion of correct predictions.

The equation for precision is as follows:

*(True positives + true negatives) / total prediction number* ^3^.

The result can be represented by a curve of performance characteristics (receiver operating characteristics (ROC) curve). This curve illustrates the relationship between the true positive rate (sensitivity) and the false positive rate (1-specificity).

To summarize the overall accuracy of the test, it is possible to calculate the area under the curve (AUC). The range of this value is between 0 and 1. A value of 0.5 indicates an absence of discrimination, a value between 0.7 and 0.8 is considered acceptable, a value between 0.8 and 0.9 is considered excellent. A value of 1 indicates perfect precision^10^.

**References:**

1. Mooney SJ, Pejaver V. Big Data in Public Health: Terminology, Machine Learning, and Privacy. *Annu Rev Public Health*. 2018;39(1):95-112. doi:10.1146/annurev-publhealth-040617-014208

2. Deo RC. Machine Learning in Medicine. *Circulation*. 2015;132(20):1920-1930. doi:10.1161/CIRCULATIONAHA.115.001593

3. Sidey-Gibbons JAM, Sidey-Gibbons CJ. Machine learning in medicine: a practical introduction. *BMC Med Res Methodol*. 2019;19(1):64. doi:10.1186/s12874-019-0681-4

4. Kriegeskorte N, Golan T. Neural network models and deep learning. *Curr Biol*. 2019;29(7):R231-R236. doi:10.1016/j.cub.2019.02.034

5. Bey R, Goussault R, Grolleau F, Benchoufi M, Porcher R. Fold-stratified cross-validation for unbiased and privacy-preserving federated learning. *Journal of the American Medical Informatics Association*. 2020;27(8):1244-1251. doi:10.1093/jamia/ocaa096

6. Le Glaz A, Haralambous Y, Kim-Dufor D-H, et al. Machine Learning and Natural Language Processing in Mental Health: Systematic Review. *J Med Internet Res*. 2021;23(5):e15708. doi:10.2196/15708

7. Mothi SS, Sudarshan M, Tandon N, et al. Machine learning improved classification of psychoses using clinical and biological stratification: Update from the bipolar-schizophrenia network for intermediate phenotypes (B-SNIP). *Schizophrenia Research*. 2019;214:60-69. doi:10.1016/j.schres.2018.04.037

8. Lin E, Lin C-H, Lane H-Y. Precision Psychiatry Applications with Pharmacogenomics: Artificial Intelligence and Machine Learning Approaches. *IJMS*. 2020;21(3):969. doi:10.3390/ijms21030969

9. Yan W, Calhoun V, Song M, et al. Discriminating schizophrenia using recurrent neural network applied on time courses of multi-site FMRI data. *EBioMedicine*. 2019;47:543-552. doi:10.1016/j.ebiom.2019.08.023

10. Mandrekar JN. Receiver operating characteristic curve in diagnostic test assessment. *J Thorac Oncol*. 2010;5(9):1315-1316. doi:10.1097/JTO.0b013e3181ec173d
